# Supplementary material for: Detailed molecular and epigenetic characterization of the pig IPEC-J2 and chicken SL-29 cell lines
Source: iScience. 2023 Feb 20;26(3):106252. doi: 10.1016/j.isci.2023.106252 (PMC10018572; doi:10.1016/j.isci.2023.106252)
Supplement: Data S2. Complete homer output for identified motifs in Chicken SL-29, related to Tables 5 and 6 — Homer motif analysis results for histone modifications H3K4me1, H3K4me3, H3K27ac, enhancers, and ATAC-seq of chicken SL-29 cell line. Parameters for possible false positives is as mentioned earlier for S5. [file mmc3.zip › Data_S2/S6/Chicken_SL_29/peak_files_CTCF/homerResults/motif6.similar.html]

motif6

## Information for motif6

A
G
C
T
T
C
G
A
G
A
C
T
A
C
T
G
C
T
G
A
A
G
T
C
C
T
A
G
G
A
C
T
T
G
A
C
C
G
T
A
  
Reverse Opposite:  

G
C
A
T
A
C
T
G
C
T
G
A
G
A
T
C
T
C
A
G
G
A
C
T
T
G
A
C
C
T
G
A
A
G
C
T
T
C
G
A
  

|  |  |
| --- | --- |
| p-value: | 1e-140 |
| log p-value: | -3.243e+02 |
| Information Content per bp: | 1.504 |
| Number of Target Sequences with motif | 2533.0 |
| Percentage of Target Sequences with motif | 8.59% |
| Number of Background Sequences with motif | 1471.1 |
| Percentage of Background Sequences with motif | 5.06% |
| Average Position of motif in Targets | 152.6 +/- 79.0bp |
| Average Position of motif in Background | 148.0 +/- 92.5bp |
| Strand Bias (log2 ratio + to - strand density) | 0.0 |
| Multiplicity (# of sites on avg that occur together) | 1.15 |
| Motif File: | file (matrix) reverse opposite |

### Similar de novo motifs found

|  |  |  |  |  |  |  |  |
| --- | --- | --- | --- | --- | --- | --- | --- |
| Rank | Match Score | Redundant Motif | P-value | log P-value | % of Targets | % of Background | Motif file |
| 1 | 0.912 | G A C T C T A G T C G A G A T C A C T G C A G T T A G C G C T A | 1e-108 | -250.810213 | 25.54% | 20.19% | motif file (matrix) |
| 2 | 0.967 | A T G C T A C G A T G C A T C G T C A G G A C T A C T G T C G A A G T C C T A G A G C T T A G C T C G A | 1e-104 | -241.666440 | 7.32% | 4.47% | motif file (matrix) |
